# Presence of CD80 and Absence of LAT in Modulating Cellular Infiltration and HSV-1 Latency

**DOI:** 10.3390/v16091379

**Published:** 2024-08-29

**Authors:** Ujjaldeep Jaggi, Homayon Ghiasi

**Affiliations:** Center for Neurobiology and Vaccine Development, Ophthalmology Research, Department of Surgery, Cedars-Sinai Burns & Allen Research Institute, CSMC – SSB3, 8700 Beverly Blvd., Los Angeles, CA 90048, USA; ujjaldeep.jaggi@cshs.org

**Keywords:** corneal scarring, CD8, PD-1, virus replication, latency, reactivation

## Abstract

CD80 is the best-known costimulatory molecule for effective T cell functions. Many different reports have summarized the role of CD80 in HSV-1 and its functions in maintaining adaptive immunity, which is the main player in causing herpes stromal keratitis (HSK). To determine the effects of absence or overexpression of CD80 in HSV-1 infection, we infected CD80^-/-^ and WT mice with a recombinant HSV-1 expressing murine CD80 (HSV-CD80) in place of the latency associated transcript (LAT). Parental dLAT2903 virus lacking LAT was used as a control. After infection, critical components of infection like virus replication, eye disease, early cellular infiltrates into the corneas and trigeminal ganglia (TG), latency-reactivation in the infected mice were determined. Our findings reveal that the absence of CD80 in the CD80^-/-^ mice infected with both viruses did not affect the viral titers in the mice eyes or eye disease, but it played a significant role in critical components of HSV-induced immunopathology. The WT mice infected with dLAT2903 virus had significantly higher levels of latency compared with the CD80^-/-^ mice infected with dLAT2903 virus, while levels of latency as determined by gB DNA expression were similar between the WT and CD80^-/-^ mice infected with HSV-CD80 virus. In contrast to the differences in the levels of latency between the infected groups, the absence of CD80 expression in the CD80^-/-^ mice or its overexpression by HSV-CD80 virus did not have any effect on the time of reactivation. Furthermore, the absence of CD80 expression contributed to more inflammation in the CD80^-/-^-infected mice. Overall, this study suggests that in the absence of CD80, inflammation increases, latency is reduced, but reactivation is not affected. Altogether, our study suggests that reduced latency correlated with reduced levels of inflammatory molecules and blocking or reducing expression of CD80 could be used to mitigate the immune responses, therefore controlling HSV-induced infection.

## 1. Introduction

HSV-1-induced eye disease can lead to blindness and is the leading cause of infectious blindness in developed countries [1]. Eye disease associated with ocular infection is mainly due to recurrent infection rather than primary infection [2]. It is well established that HSV-1-induced eye disease is a result of immune responses triggered by the virus [3,4,5]. Adoptive transfer and in vivo T-cell subset depletion studies have suggested that CD8^+^ T cells alone [6,7,8,9], CD4^+^ T cells alone [10,11,12,13], or both together [10,14,15] contribute to HSV-1-induced eye disease. T cells require two signals to become fully activated [16]; the first signal is antigen (Ag)-specific, while the second signal is generated by the binding of CD28 on the T cells to CD80 (B7-1) or CD86 (B7-2) on antigen-presenting cells [17]. CD28–B7 interaction leads to T cell proliferation, differentiation, and cytokine secretion [18,19]. Previously, we have shown that ICP22 but not any other HSV-1 genes repress CD80 but not CD86 expression by directly binding to the CD80 promoter [20]. The ability of ICP22 to interact with and suppress CD80 dampens the host immune response, allowing HSV-1 to partially escape immune surveillance, leading to reduced eye disease [21]. Thus, ICP22 may be a novel CD80 inhibitor that could be used therapeutically to modulate the immune responses. The precise biological function of ICP22 is unknown, but our published study suggests that mice infected with a recombinant HSV-1 expressing CD80 have elevated CD80 and CD8 and enhanced corneal scaring (CS) [20,21]. We have also shown that the absence of ICP22 enhances eye disease in ocularly infected mice [21]. Since down-regulation of CD80 and CD8 is required for virus infectivity, HSV-1 may use ICP22 as a survival mechanism by reducing the CTL function of CD8, thus blocking cell lysis. We have also shown that a recombinant HSV-1 expressing CD80 exacerbated CS in infected BALB/c and C57BL/6 mice [20,22]. As a proof-of-principal, we have shown that mice ocularly infected with a recombinant HSV-1 lacking ICP22 developed enhanced eye disease [21]. Furthermore, we have shown that using CD80 in place of LAT compensated for the latency-reactivation and anti-apoptotic functions of LAT [22].

Two main factors that control the adaptive immune phase are the CD80-CD86 costimulatory molecules, which lead to the T-cell activation and proliferation that drive the initiation of adaptive immunity [19]. However, our published studies have shown that CD80 but not CD86 plays a critical role in increased inflammatory responses in HSV-1-infected mouse corneas [20]. In the current study, we looked at how the presence or absence of CD80 and LAT as well as overexpression of CD80 using a recombinant virus expressing murine CD80 (HSV-CD80) would affect HSV-1 infectivity by comparing CD80^-/-^ mice with WT control mice infected with HSV-CD80 or dLAT2903 (LAT-minus) virus. Our results suggest that there were no significant differences in the eye disease or virus replication in the eyes between the two mice groups infected with HSV-CD80 and dLAT2903 viruses, but in the absence of CD80 in the CD80^-/-^ mice with dLAT2903 virus infection, inflammation increased with enhanced IFN-ϒ response. Virus infectivity as measured with the gB transcript was higher in the WT mice infected with dLAT2903 virus in comparison to the CD80^-/-^ mice infected with dLAT2903, which suggests that the absence of CD80 in dLAT2903 virus increases virus infectivity. CTLA4 gene expression along with CD4^+^ T cells was significantly enhanced in the WT mice infected with dLAT2903 virus as compared to the CD80^-/-^ mice infected with HSV-CD80 virus. Overall, our results show that CD80 does not interfere with disease progression or reactivation, but its absence in the host helps to control virus infectivity and thus minimizing establishment of latency, thereby aiding in improving the survival of the host.

## 2. Materials and Methods

**Ethics statement.** All animal procedures were performed in strict accordance with the Association for Research in Vision and Ophthalmology Statement for the Use of Animals in Ophthalmic and Vision Research and the NIH *Guide for the Care and Use of Laboratory Animals* (ISBN 0-309-05377-3). Animal research protocols were approved by the Institutional Animal Care and Use Committee of Cedars-Sinai Medical Center (Protocol #8837).

**Mice.** Wild-type (WT) C57BL/6 and C57BL/6-CD80^-/-^ mice were purchased from Jackson Laboratory and were bred and maintained in the Cedars-Sinai Medical Center pathogen–free animal facility. Six–eight-week-old male and female WT and CD80^-/-^ mice were used in the study.

**Viruses and cells.** Triply plaque-purified HSV-CD80 and dLAT2903 (parental virus) HSV-1 strain were used for all experiments in this study. dLAT2903 and HSV-CD80 viruses were described previously [23,24]. Viruses were grown in rabbit skin (RS) cell monolayers in minimal essential medium (MEM) containing 5% fetal calf serum (FCS), as we described previously [23,24].

**Ocular infection.** WT and CD80^-/-^ mice were infected with 2 × 10^5^ PFU/eye of HSV-CD80 and dLAT2903 McKrae viruses as an eye drop in 2 μL of tissue culture media as we described previously [25]. Corneal scarification was not performed prior to infection.

**Viral titers from tears of infected mice.** Tear films were collected from twenty-two mice eyes per group on days 1–7 post infection (PI) using a Dacron-tipped swab. Each swab was placed in 1 mL of tissue culture medium and squeezed. The amount of virus was determined using a standard plaque assay on RS cells as described [21].

**Monitoring corneal scarring.** The severity of CS lesions in mouse corneas was examined by slit lamp biomicroscopy using a scoring scale of 0, normal cornea; 1, mild haze; 2, moderate opacity; 3, severe corneal opacity but iris visible; 4, opaque and corneal ulcer; 5, corneal rupture and necrotizing keratitis.

**In vitro explant reactivation assay.** Mice were sacrificed on day 28 PI and individual TG were removed and cultured in tissue culture media as described [22]. Media aliquots were removed from each culture daily and plated on RS indicator cells to detect reactivated virus and to determine the time at which reactivated virus first appeared in the explanted TG cultures.

**RNA and DNA extraction, cDNA synthesis, TaqMan PCR, and RT-PCR.** Corneas and TG from individual mice were isolated on days 3, 5, and 7 PI, while on day 28 PI, TG from latently infected mice were collected for RNA extraction, DNA extraction, or reactivation. Collected tissues were processed as described previously [26]. Expressions of LAT RNA from latent TG were determined using custom-made LAT primers and probe as follows: forward primer, 5′-GGGTGGGCTCGTGTTACAG-3′; reverse primer, 5′-GGACGGGTAAGTAACAGAGTCTCTA-3′; and probe, 5′-FAM-ACACCAGCCCGTTC

TTT-3′ (amplicon length = 81 bp). Levels of gB DNA in latent TG was isolated from homogenized individual TG using the commercially available Dnaeasy Blood &Tissue Kit (Qiagen, Stanford, CA) according to the manufacturer’s instructions. PCR analyses were performed using gB specific primers: (forward primer, 5’-AACGCGACGCACATCAAG-3’; reverse primer, 5’-CTGGTACGCGATCAGAAAGC-3’; and probe—5’-FAM-CAGCCGCAGTACTACC-3’ (amplicon length = 72 bp). The relative copy numbers of LAT RNA and gB DNA were calculated using standard curves generated from plasmids pGem5317 and pAc-gB1, respectively, by comparing the normalized threshold cycle (*C_T_*) of each sample to the threshold cycle of the standard curve.

Expressions of primary (days 3, 5, and 7) and latent (day 28) genes were measured using qRT-PCR as follows: (1) CD4 (ABI Mm00442754_m1; amplicon length = 72 bp); (2) CD8α (ABI Mm01182108_m1; amplicon length = 67 bp); (3) F4/80 (Mm00802529_m1; amplicon length = 92 bp); (4) CD11c (Mm00498701_m1; amplicon length = 93 bp); (5) Ly6G (Mm04934123_m1; amplicon length = 113 bp); (6) NK1.1 (Mm00824341_m1; amplicon length = 92 bp); (7) IL-2 (Mm00434256_m1; amplicon length = 82 bp); (8) IL-4 (Mm00445259_m1; amplicon length = 79 bp); (9) IL-6 (Mm00446190_m1; amplicon length = 78 bp); (10) IFN-γ (Mm00801778_m1; amplicon length = 101 bp); (11) IFN-α2A (Mm00833961_s1; amplicon length = 158 bp); (12) IFN-β (Mm00439552_s1; amplicon length = 69 bp); (13) CD80 (MM00711660_m1; amplicon length = 117 bp); (14) CD86 (Mm00444540_m1; amplicon length = 91 bp); (15) CD28 (Mm01253994_m1; amplicon length = 98 bp); (16) PD-L1 (Mm03048248_m1; amplicon length = 73 bp); (17) CTLA4 (Mm00486849_m1; amplicon length = 71 bp); (18) IL-1α (Mm00439620_m1; amplicon length = 68 bp); (19) IL-1β (Mm00434228_m1; amplicon length = 90 bp); (20) GzmA (Mm01304452_m1; amplicon length = 59 bp); (21) GzmB (Mm00442837_m1; amplicon length = 82 bp); (22) Perforin (Mm00812512_m1; amplicon length = 95 bp); (23) TNFα (Mm00443258_m1; amplicon length = 81 bp); (24) CD45 (Mm01293577_m1; amplicon length = 73 bp); (25) IL-12α (Mm00434169_m1; amplicon length = 58 bp); (26) IL-12β (Mm99999067_m1; amplicon length = 63 bp); (27) CD1d Mm00783541_s1; amplicon length = 142 bp); and (28) PD-1 (programmed death 1; ABI Mm00435532_m1; amplicon length = 65 bp). GAPDH served as an internal control in all experiments as (Mm99999915_g1; amplicon length = 107 bp) to normalize transcripts. Transcripts in corneas and TG were evaluated on different days in acute and latent stages of infection using commercially available TaqMan Gene Expression Assays (Applied Biosystems, Foster City, CA, USA) with optimized primer and probe concentrations. The 2^−ΔΔ*CT*^ method was used to calculate fold changes in gene expressions relative to expressions in uninfected controls.

**Statistical analysis.** For all statistical tests, *p*-values less than or equal to 0.05 were considered statistically significant and are indicated by a single asterisk (*). *p*-values less than or equal to 0.001 are indicated by double asterisks (**). A two-tailed Student’s *t*-test with unequal variances was used to compare differences between two experimental groups. A one-way ANOVA test was used to compare differences among three or more experimental groups. All experiments were repeated at least two times to ensure accuracy.

## 3. Results

**Figure 1:**
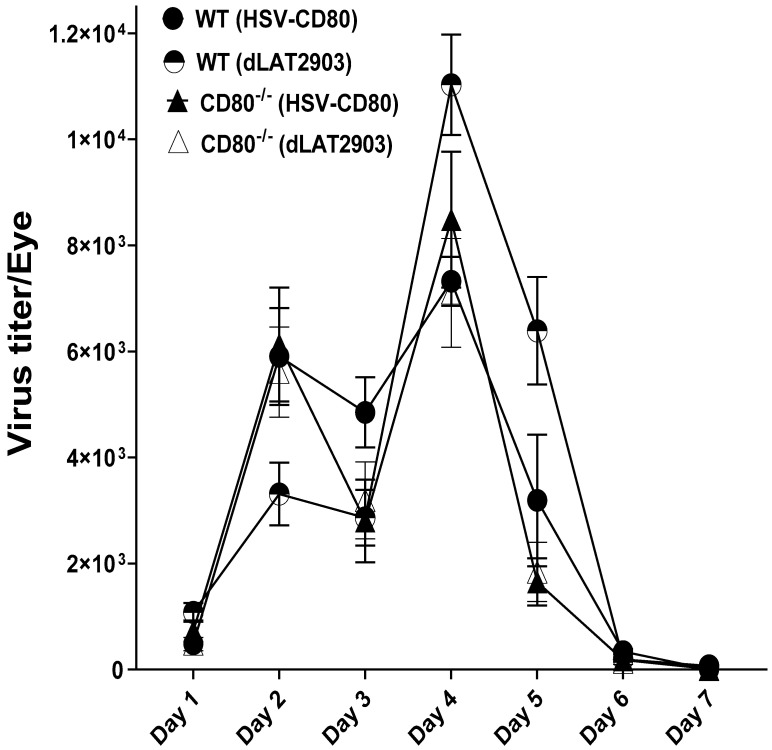
**Viral titers in WT and CD80^-/-^ mice eyes following ocular infection with dLAT2903 and HSV-CD80 viruses.** WT and CD80^-/-^ mice were infected with 2 × 10^5^ PFU/eye of dLAT2903 and HSV-CD80 viruses. The presence of infectious virus in the eyes of infected mice was monitored daily for 7 days by collecting tear films and quantifying the virus using standard plaque assays as described (see Section 4). Each point represents the mean ± SEM from 24 eyes for all infected mouse groups; no differences in viral titers were seen among the four groups. The experiment was repeated twice. The levels of virus shedding were not significantly different in the respective groups.

**CD80 absence or overexpression does not alter virus replication in the eyes of infected mice.** Our previous findings demonstrate that CD80 overexpression does not affect virus replication [27], but CD80 absence reduces virus replication [28]. Therefore, to test the effect of CD80 in our current study, we ocularly infected CD80^-/-^ and WT mice with 2 × 10^5^ PFU/eye of HSV-CD80 and dLAT2903 (parental virus) viruses. Tear films were collected by eye swabs on days 1–7 PI from 24 eyes of the WT and CD80^-/-^ mice infected with HSV-CD80 or dLAT2903 virus. Virus titers were determined by standard plaque assays in all of the four infected mice groups. Virus titers on day 2 PI in the eyes of dLAT2903-infected WT mice were reduced as compared with all three other infected mice groups, but the differences were not statistically significant (Figure 1, *p* > 0.05). By day 4 PI, virus titers in all four infected mice groups peaked, thereafter declining on days 6–7 PI (Figure 1, *p* > 0.05). These results suggest that the absence of CD80 in the CD80^-/-^ mice infected with dLAT2903 virus was reduced as compared to the other groups, and this reduced virus replication in the eyes of infected mice could play a critical role in HSV-1 pathogenesis. However, virus replication in CD80^-/-^ mice after infection with HSV-CD80 virus was similar to that in the WT-infected group, suggesting that the absence of CD80 in CD80^-/-^ mice was compensated by expression of CD80 by HSV-CD80 virus, which increased viral titers in the infected eyes of CD80^-/-^ mice, but not in those of the WT-infected mice.

**Figure 2:**
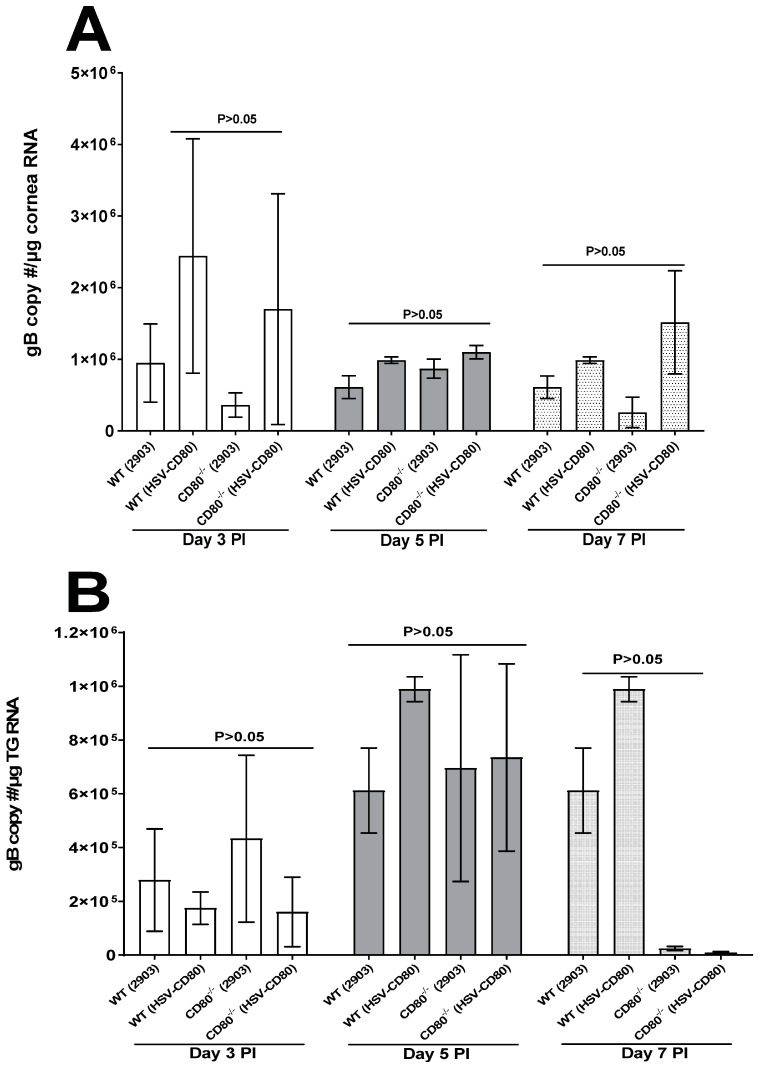
**Quantification of the gB copy numbers in the corneas and TG of the infected WT and CD80^-/-^ mice.** (**A**) The gB copy numbers in the infected corneas. WT and CD80^-/-^ mice were infected with 2 × 10^5^ PFU/eye of dLAT2903 and HSV-CD80 viruses, and the corneas and TG from the WT- and CD80^-/-^-infected mice (3 mice/group) were harvested on days 3, 5, and 7 PI. The total RNA was isolated from each cornea or TG, and GAPDH expression was used to normalize the expression of the gB transcripts in the corneas or TG of the infected mice, and the gB copy numbers were determined by qRT-PCR as described in Section 2. No differences were observed in the infected corneas between the four infected mice groups (*p* > 0.05). Each bar represents the mean expression ± SEM in the six corneas from all the infected mouse groups; and (**B**) the gB copy numbers in the infected TG. The TG were harvested on days 3, 5, and 7 PI from the above infected mice. The total RNA was isolated from each TG, and GAPDH expression was used to normalize the expression of each transcript in the TG of ocularly infected mice, and the gB copy numbers were determined as above. No differences in the infected TG were observed between the four infected mice groups (*p* > 0.05). Each bar represents the mean expression ± SEM in the six TG from all the infected mouse groups. Only the differences that are statistically significant are shown for each gene.

We next investigated viral glycoprotein gB expression as an indicator of HSV-1 replication using qRT-PCR. WT and CD80^-/-^ mice were infected with HSV-CD80 or dLAT2903 virus as above. Corneas and TG from the infected mice were isolated on days 3, 5, and 7 PI, and the total RNA was isolated as described in Section 2. We found no significant differences in gB expression levels on days 3, 5, and 7 between the CD80^-/-^ and WT mice infected with the two viruses (Figure 2A,B, *p* > 0.05). However, the gB copy numbers in both the corneas and TG followed a lower trend in the absence of CD80 in the CD80^-/-^ mice infected with dLAT2903 virus (Figure 2A,B, *p* > 0.05).

**Host immune response after infection in controlling virus replication.** In one of our published reports, we showed the effect of HSV-1 infection leading to a cytokine storm [29]. Herein, we used a different technique to measure all the host factors that could potentially be affected by HSV-1 infection using a customized panel that included twenty-eight genes in addition to control genes and performed qRT-PCR as described in Section 2. We investigated the roles of T cells (CD4^+^ and CD8^+^), innate immune cells (F4/80, CD11c, NK1.1, and Ly6G), cytokines (IL-1α, IL-1β, IL-2, IL-4, IL-6, IFNα2, IFNβ, IFNγ, IL-12α, and IL-12β), costimulatory molecules (CD80, CD86, CD28, CTLA4, and CD1d), immune-mediated cytotoxicity molecules (perforin and granzyme A and B), PD-L1, CD45, and TNF-α. To perform this study, WT and CD80^-/-^ mice were ocularly infected with 2 × 10^5^ PFU/eye of HSV-CD80 and dLAT2903 viruses. On days 3, 5, and 7 PI, the corneas and TG were extracted, and the total RNA was isolated as above. In both the corneas and TG, the expression levels of Ly6G, IL-2, IL-4, IFNα2, IFNβ1, IL-12α, IL-12β, CD28, and CD1d were not statistically significant on days 3, 5, and 7.

**Figure 3:**
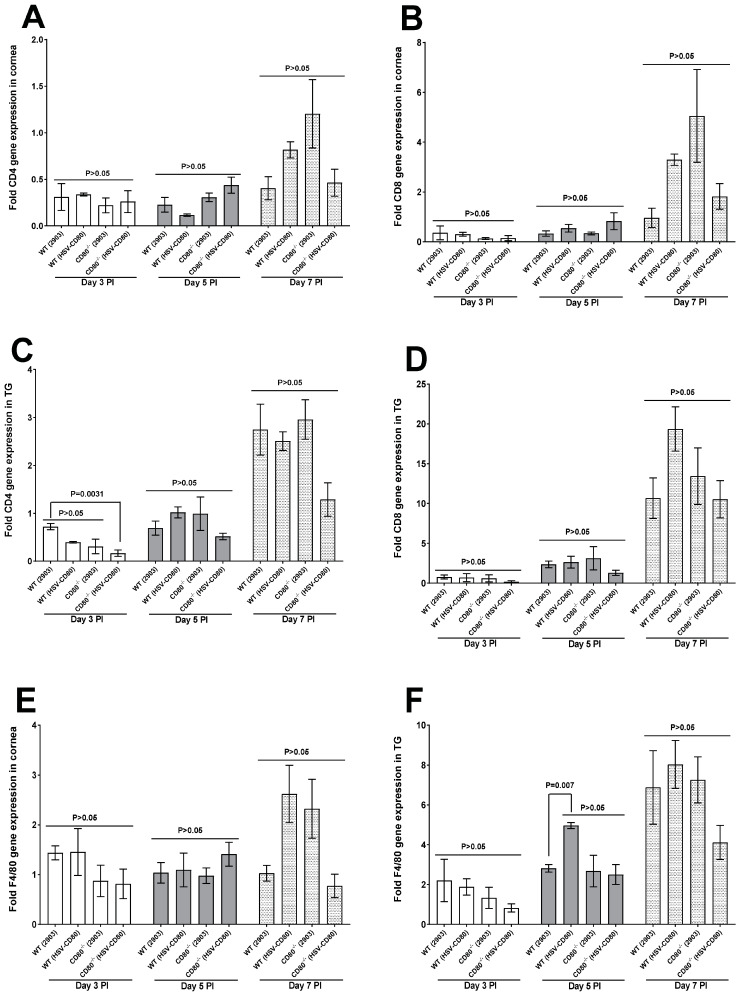
**Quantification of CD4, CD8α, and F4/80 RNA transcripts in the corneas and TG of the infected WT and CD80^-/-^ mice**. (**A**,**B**) Expressions of CD4 and CD8α in the infected corneas. WT and CD80^-/-^ mice were infected as described in Figure 2 above. The total RNA was isolated from each cornea, and GAPDH expression was used to normalize the expressions of CD4 and CD8α transcripts in the corneas of ocularly infected mice. CD4 and CD8α in the infected corneas displayed no significant differences on days 3, 5 and 7 PI among all the infected mice groups (*p* > 0.05); (**C**,**D**) expression of CD4 and CD8α in the infected TG. The total RNA was isolated from each TG, and GAPDH expression was used to normalize the expressions of CD4 and CD8α transcripts in the TG of ocularly infected mice. CD4 T cells transcript levels on day 3 PI in the infected TG in the WT mice infected with dLAT2903 virus displayed higher expression levels in comparison to CD4 transcript levels in CD80^-/-^ mice infected with HSV-CD80 virus (*p* < 0.0031). CD8α T cells in the TG were not significantly different in any of the infected mice groups (*p* > 0.05); and (**E**,**F**) expression levels of F4/80 RNA transcripts in the corneas and TG. The total RNA was isolated from each cornea or TG, and GAPDH expression was used to normalize the expression levels of F4/80 transcripts in the corneas and TG of ocularly infected mice. F4/80 expression levels in the infected corneas displayed no significant differences on days 3, 5 and 7 PI among all the infected groups (*p* > 0.05). F4/80 expression levels in the infected TG were higher on day 5 PI in the WT mice infected with HSV-CD80 virus as compared with the WT mice infected with dLAT2903 virus (*p* = 0.0067). Each bar represents the mean expression ± SEM in the 6 Corneas and TG from all the infected mouse groups.

**Innate immune cell markers:** Innate immunity marks the hallmark for any viral infection as these are the first responders to infection. To determine the effectiveness of innate immunity to HSV infection, we evaluated the expression levels of the most critical cell components like macrophages (F4/80), dendritic cells (CD11c), and Natural killer cells (NK1.1) in the corneas and TG of WT- and CD80^-/-^-infected mice. No differences in the expression levels of F4/80 were observed in the corneas of the four infected groups, with minimal expression on days 3 and 5 PI and higher expression levels on day 7 PI (Figure 3E, *p* > 0.05). We also examined infected TG in WT and CD80^-/-^ mice using the same approach and found that F4/80 expression was higher in the WT mice infected with HSV-CD80 virus as compared to the WT mice infected with dLAT2903 virus (Figure 3F, *p* = 0.007), with no significant differences among other days at any point (Figure 3F, *p* > 0.05).

**Figure 4:**
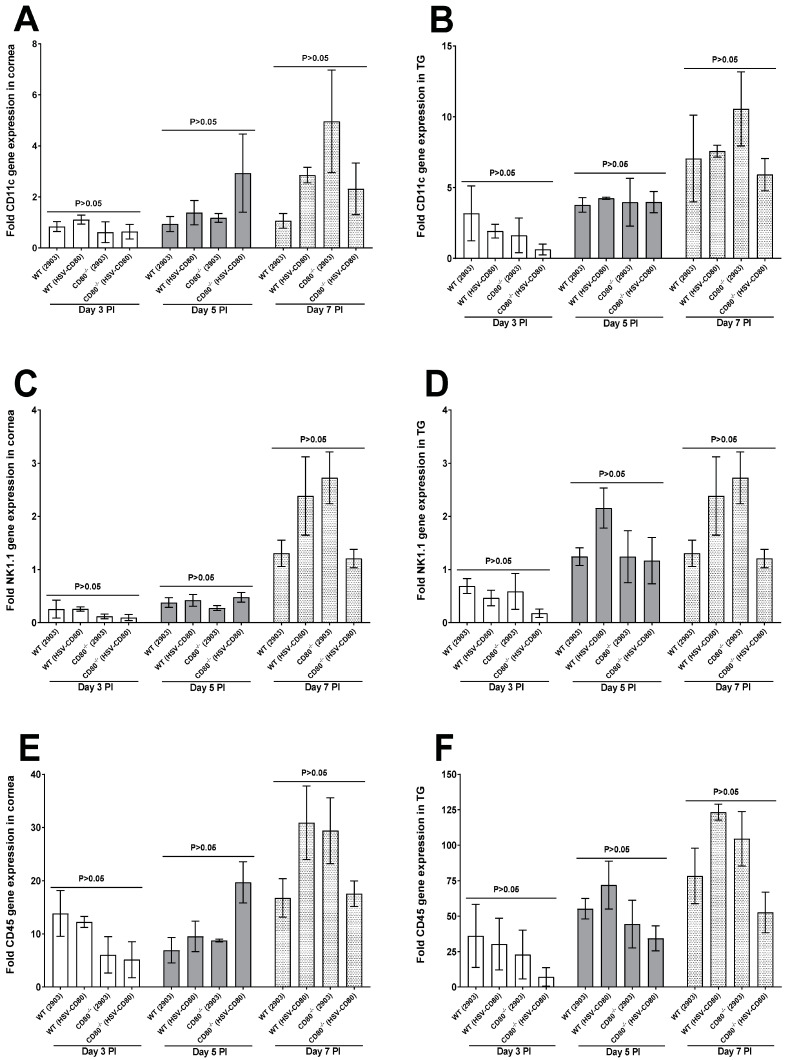
**Quantification of CD11c, NK1.1, and CD45 RNA transcripts in the corneas and TG of the infected WT and CD80^-/-^ mice.** (**A**,**B**) Expression levels of CD11c in the infected corneas and TG. WT and CD80^-/-^ mice were infected as described in Figure 2. The total RNA was isolated from each cornea and TG, and GAPDH expression was used to normalize expression of the CD11c transcript in the corneas and TG of the ocularly infected mice. No significant expression level differences were detected for CD11c in the infected mouse groups; (**C**,**D**) expression levels of NK1.1 in the infected corneas and TG. The total RNA was isolated from each cornea and TG, and GAPDH expression was used to normalize expression of the NK1.1 transcript in the ocularly infected mice. No significant expression level differences were detected for NK1.1 in the infected mouse groups; and (**E**,**F**) expression levels of CD45 in the infected corneas and TG. The total RNA was isolated from each cornea and TG, and GAPDH expression was used to normalize expression of the CD45 transcript of the ocularly infected mice. No significant expression level differences were detected for CD45 in the infected mouse groups. Each bar represents the mean expression ± SEM in the six corneas and TG for all the infected mouse groups. Only the differences that are statistically significant are shown for each gene.

We previously showed that HSV-1 downregulates CD80 expression by DCs and not any other cell type [20]. To evaluate our finding in our current model, we examined CD11c expression levels in corneas and TG of infected mice. There were no significant differences among the groups in the corneas or TG of the infected mice; however, CD11c expression levels followed a higher trend in the CD80^-/-^ mice infected with dLAT2903 virus in comparison to other groups (Figure 4A,B, *p* > 0.05). The trend was similar with regards to expressions of NK1.1 and CD45 in both the corneas and TG of the infected mice (Figure 4C–F, *p* > 0.05).

**T cells:** We selected CD4 and CD8α T cells expressions to screen since T cells are known to exacerbate HSV-1 infection [30]. Both CD4 and CD8α T cells in the corneas of the infected mice followed a reduced trend although not statistically different on days 3 and 5 PI but followed an increased expression trend on day 7 PI in both the WT- and CD80^-/-^-infected mice, especially in the CD80^-/-^ mice infected with dLAT2903 virus, which was not significantly different but shows the absence of CD80 does not alter CD4 and CD8α T cells expression (Figure 3A,B, *p* > 0.05). Similarly, we investigated the effects of CD4 and CD8α in the TG of the infected mice. CD4 T cells in the TG of the WT mice infected with dLAT2903 virus were significantly increased in comparison to CD4 T cells in the TG of the CD80^-/-^ mice infected with HSV-CD80 virus (Figure 3C, *p* = 0.0031). On the other hand, CD8α T cells showed no statistical differences among any infected mice groups but again followed an increased non-significant expression trend on day 5 PI, whereas on days 3 and 5 PI the CD8α T cells expression levels were almost negligible (Figure 3D, *p* > 0.05). This shows that CD80 suppresses immune cells as a means of immune escape, as shown before [21].

**Figure 5:**
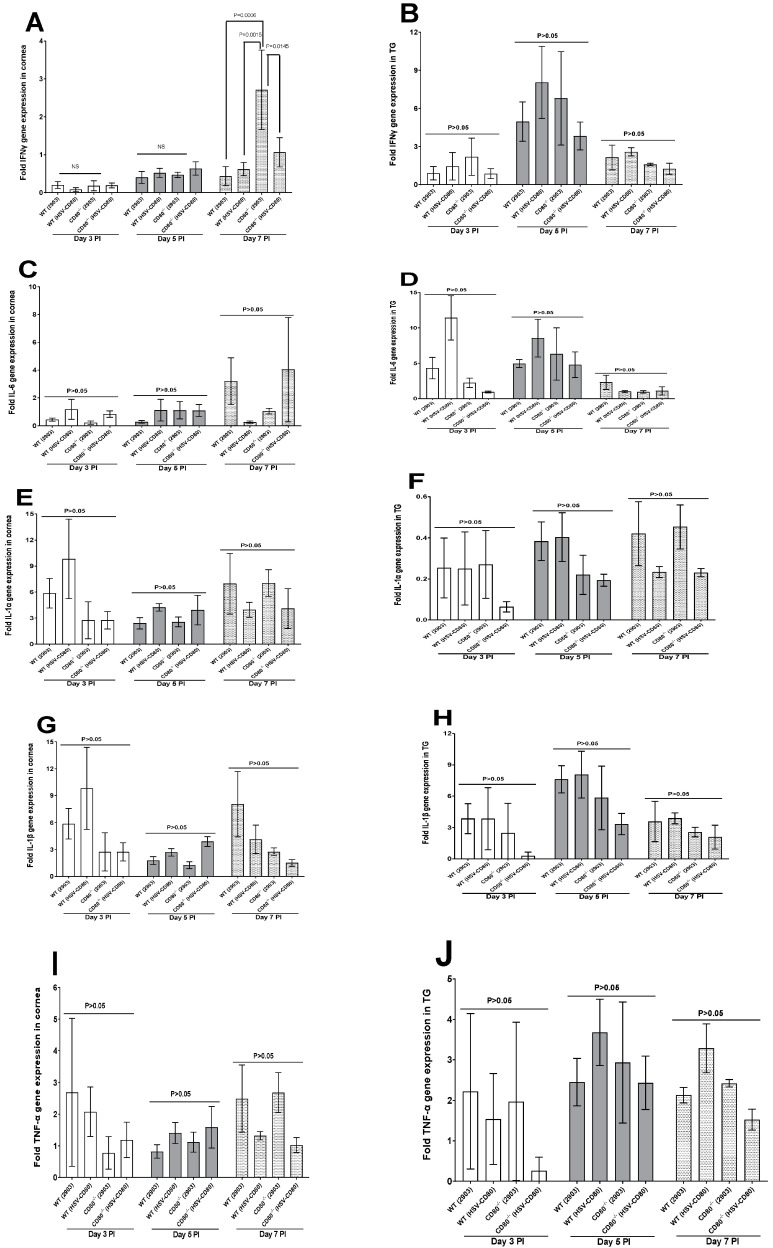
**Quantification of IFN-γ, IL-6, IL-1α, IL-1β, and TNF-α RNA transcripts in the corneas and TG of the infected WT and CD80^-/-^ mice.** (**A**,**B**) Expression levels of IFN-γ in the infected corneas and TG. WT and CD80^-/-^ mice were infected as described in Figure 2. The total RNA was isolated from each cornea and TG. GAPDH expression was used to normalize expression levels of IFN-γ transcripts in the corneas and TG of the ocularly infected mice. The corneas from the CD80^-/-^ mice infected with dLAT2903 virus showed significantly higher expression levels of IFN-γ transcripts on day 7 PI in comparison to all three other infected mice groups (*p* < 0.05), whereas in the TG, IFN-γ transcript levels had no significant differences at any point of the day among the four groups (*p* > 0.05); (**C**,**D**) expression levels of IL-6 in the infected corneas and TG. WT and CD80^-/-^ mice were infected as described in Figure 2. The total RNA was isolated from each cornea and TG. GAPDH expression was used to normalize expression levels of the IL-6 transcript in the corneas and TG of the ocularly infected mice. The infected WT and CD80^-/-^ mice had no significant differences in IL-6 expression levels in the corneas or TG (*p* > 0.05); (**E**,**F**) expression levels of IL-1α in the infected corneas and TG. WT and CD80^-/-^ mice were infected as described in Figure 2. The total RNA was isolated from each cornea and TG, and GAPDH expression was used to normalize expression levels of the IL-1α transcript in the corneas and TG of the ocularly infected mice. The infected WT and CD80^-/-^ mice had no significant differences in IL-1α transcript expression levels in the corneas or TG (*p* > 0.05); (**G**,**H**) expression levels of IL-1β in the infected corneas and TG. WT and CD80^-/-^ mice were infected as described in Figure 2. The total RNA was isolated from each cornea and TG. GAPDH expression was used to normalize expression levels of the IL-1β transcript in the corneas and TG of the ocularly infected mice. The infected WT and CD80^-/-^ mice had no significant differences in IL-1β transcript expression levels in the corneas or TG (*p* > 0.05); and (**I**,**J**) expression levels of TNF-α in the infected corneas and TG. WT and CD80^-/-^ mice were infected as described in Figure 2. The total RNA was isolated from each cornea and TG. GAPDH expression was used to normalize expression levels of the TNF-α transcript in the corneas and TG of the ocularly infected mice. The infected WT and CD80^-/-^ mice had no significant differences in TNF-α transcript expression levels in the corneas or TG (*p* > 0.05). Each bar represents mean expression ± SEM in the six corneas and TG from all the infected mouse groups. Only the differences that are statistically significant are shown for each gene. Panels: (**A**,**B**) Expression levels of IFN-γ in the corneas and TG. (**C**,**D**) Expression levels of IL-6 in the infected corneas and TG. (**E**,**F**) Expression levels of IL-1α RNA transcripts in the corneas and TG. (**G**,**H**) Expression levels of IL-1β RNA transcripts in the infected corneas and TG. (**I**,**J**) Expression levels of TNF-α in the infected corneas and TG.

**Cytokine expression:** Since we detected immune cell infiltration into the infected corneas and TG, we next investigated possible inflammatory/anti-inflammatory cytokine expressions in the corneas and TG of WT and CD80^-/-^ mice infected with HSV-CD80 or dLAT2903 virus. Using the same panel as above, IFN-γ, IL-6, IL-1α, IL-1β, and CD45 were measured in both the corneas and TG of the infected mice on days 3, 5 and 7 PI. The expression levels of IFN-γ in the infected corneas were negligible on days 3 and 5 PI, but on day 7 PI, the CD80^-/-^ mice infected with dLAT2903 virus showed a significant increase in IFN-γ expression as compared with all three other groups {(Figure 5A; CD80^-/-^ (dLAT2903) vs. WT (dLAT2903), *p* = 0.0006, CD80^-/-^ (dLAT2903) vs. WT (HSV-CD80), *p* = 0.0015 and CD80^-/-^ (dLAT2903) vs. CD80^-/-^ (HSV-CD80), *p* = 0.0145}. However, the IFN-γ detected in the TG of the infected mice did not differ among the four groups (Figure 5B, *p* > 0.05), and so was the case with IL-6, IL-1α, IL-1β, and CD45 (Figure 5C–G,I, *p* > 0.05).

**Figure 6:**
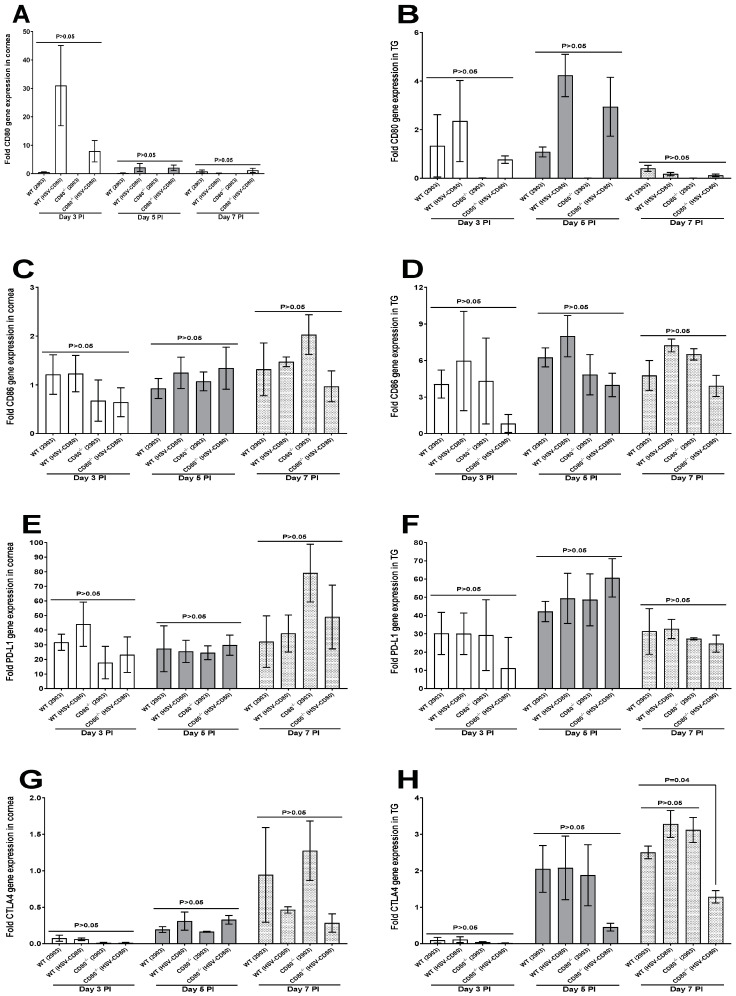
**CD80, CD86, PD-L1, and CTLA4 expressions in the corneas and TG of the infected WT and CD80^-/-^ mice.** (**A**,**B**) Expression levels of CD80 in the infected corneas and TG. WT and CD80^-/-^ mice were infected as described in Figure 2. The total RNA was isolated from each cornea and TG. GAPDH expression was used to normalize expression levels of CD80 transcripts in the corneas and TG of the ocularly infected mice. CD80 transcript levels had no significant differences at any point of the day (*p* > 0.05); (**C**,**D**) expression levels of CD86 in the infected corneas and TG. WT and CD80^-/-^ mice were infected as described in Figure 2. The total RNA was isolated from each cornea and TG. GAPDH expression was used to normalize expression levels of CD86 transcripts in the corneas and TG of the ocularly infected mice. CD86 transcript levels had no significant differences at any point of the day or in any infected mice groups (*p* > 0.05); (**E**,**F**) expression levels of PD-L1 in the infected corneas and TG. WT and CD80^-/-^ mice were infected as described in Figure 2. The total RNA was isolated from each cornea and TG. GAPDH expression was used to normalize expression levels of PD-L1 transcripts in the corneas and TG of the ocularly infected mice. PD-L1 transcript levels had no significant differences at any point of the day or in any infected mice groups (*p* > 0.05); and (**G**,**H**) expression levels of CTLA4 in the infected corneas and TG. WT and CD80^-/-^ mice were infected as described in Figure 2. The total RNA was isolated from each cornea and TG. GAPDH expression was used to normalize expression levels of CTLA4 transcripts in the corneas and TG of the ocularly infected mice. CTLA4 transcript levels in the corneas had no significant differences at any point of the day or in any infected mice groups (*p* > 0.05), but in the TG on day 7 PI, CTLA4 in the WT (dLAT2903 virus-infected) mice was higher as compared with the CD80^-/-^ (HSV-CD80 virus-infected) mice. Each bar represents mean expression ± SEM in the six corneas and TG from all the infected mouse groups. Only the differences that are statistically significant are shown for each gene.

In the same experimental set up, we also investigated expression levels of costimulatory CD80 and CD86 and also T cell exhaustion markers (PD-L1 and CTLA4) by qRT-PCR using the same customized TaqMan assay plates. The CD80^-/-^ mice infected with dLAT2903 virus had no CD80 expression in their corneas on days 3, 5 and 7 PI, while CD80 expression was detected in the CD80^-/-^ mice infected with HSV-CD80 virus (Figure 6A, *p* > 0.05). The WT mice infected with HSV-CD80 virus had the highest levels of CD80 expression compared with the WT mice infected with dLAT2903 virus; however, the differences were not statistically significant (Figure 6A, *p* > 0.05). A similar pattern was detected in the TG of the infected mice, where CD80 was expressed in the WT mice but not in the CD80^-/-^ mice infected with dLAT2903 virus (Figure 6B, *p* > 0.05). On evaluating CD86 and PDL-1 expression levels in the infected corneas or TG, we found no differences on any day at any point (Figure 6C–F, *p* > 0.05). CTLA4 expression levels were negligible on day 3 PI in the infected corneas of all four groups but increased by day 7 PI, but the differences were not statistically significant (Figure 6G, *p* > 0.05). A similar trend was observed with regards to CTLA4 expression levels in the TG, but on day 7 PI, the levels of CTLA4 in the WT mice infected with dLAT2903 virus was significantly higher as compared to those of the CD80^-/-^ mice infected with HSV-CD80 virus, with no differences in other infected mice groups (Figure 6H, *p* = 0.04). Our results suggest that CD80 does not alter expression of CD4 in the TG.

**Figure 7:**
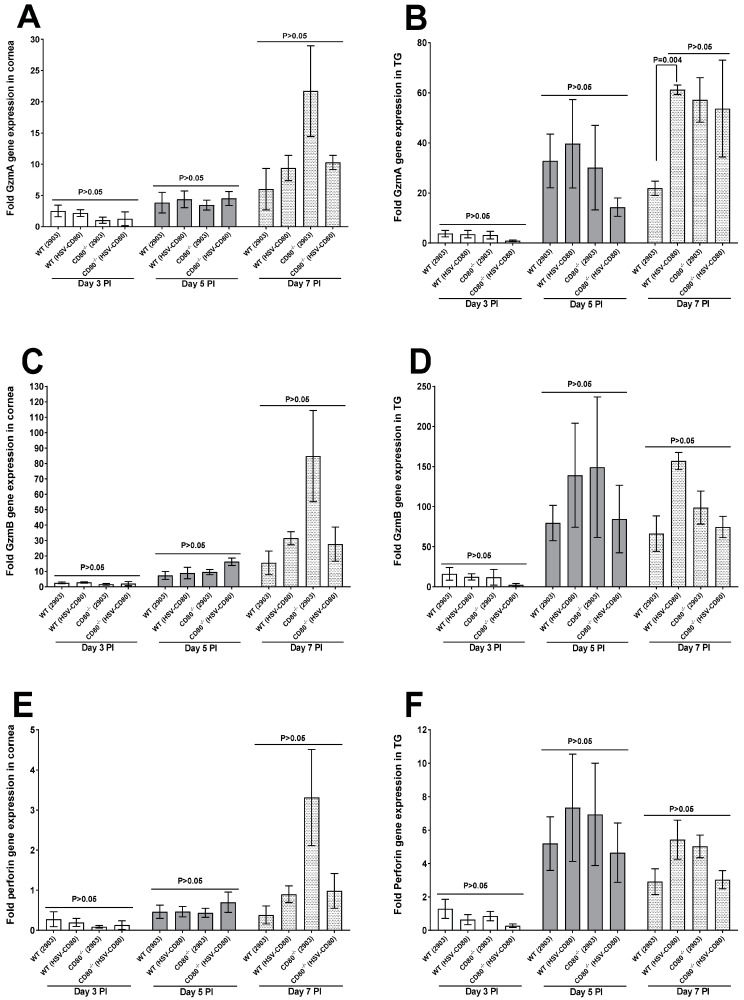
**GzmA, GzmB, and perforin expressions in the corneas and TG of the infected WT and CD80^-/-^ mice.** (**A**,**B**) Expression levels of GzmA in the infected corneas and TG. WT and CD80^-/-^ mice were infected as described in Figure 2. The total RNA was isolated from each cornea and TG. GAPDH expression was used to normalize expression levels of GzmA transcripts in the corneas and TG of the ocularly infected mice. Where GzmA transcript levels in the corneas had no significant differences, GzmA in the TG on day 7 PI was higher in the WT (HSV-CD80 virus-infected) mice as compared to the WT (dLAT2903 virus-infected) mice; (**C**,**D**) expression levels of GzmB in the infected corneas and TG. WT and CD80^-/-^ mice were infected as described in Figure 2. The total RNA was isolated from each cornea and TG. GAPDH expression was used to normalize expression levels of GzmB transcripts in the corneas and TG of the ocularly infected mice. GzmB transcript levels in the corneas and TG had no significant differences in all the infected mice groups (*p* > 0.05); and (**E**,**F**) expression levels of Perforin in the infected corneas and TG. WT and CD80^-/-^ mice were infected as described in Figure 2. The total RNA was isolated from each cornea and TG. GAPDH expression was used to normalize expression levels of perforin transcripts in the corneas and TG of the ocularly infected mice. Perforin transcript levels had no significant differences at any point of the day (*p* > 0.05).

**Tryptase and Protease molecules:** In the last customized panel, the possible effects of granzyme A (a tryptase), granzyme B (a serine protease), and perforin were investigated since cytotoxic T lymphocytes and NK cells release granules containing perforin and granzymes at target cells [31]. No significant expressions of GzmA were observed in the corneas of the infected mice on day 3, 5 or 7 PI (Figure 7A, *p* > 0.05), but on day 7 PI, GzmA expression was significantly increased in the TG of the WT mice infected with HSV-CD80 virus in comparison to the WT mice infected with dLAT2903 virus (Figure 7B, *p* = 0.004). GzmB and perforin had no significant differences in their expression levels in the infected mice (Figure 7C–F, *p* > 0.05).

Our data with regards to immune cells profiling signifies that the absence or overexpression of CD80 maintains the cellular homeostasis in infected corneas but changes the immune profiling in infected TG, supporting the role of CD80 in maintaining tissue latency. We observed that in the presence of CD80 in WT mice infected with dLAT2903 virus, both CD4 and CTLA4 expressions were upregulated. This is in line with previous research showing that CD80 binding is required for CTLA4 expression and to keep T cells functions in check [32]. Furthermore, our results have also shown that overexpression of CD80 in WT mice infected with HSV-CD80 virus leads to increased expression levels of F4/80. This is also similar to a previous study that showed CD80 is required for activation of macrophages [33].

**Figure 8:**
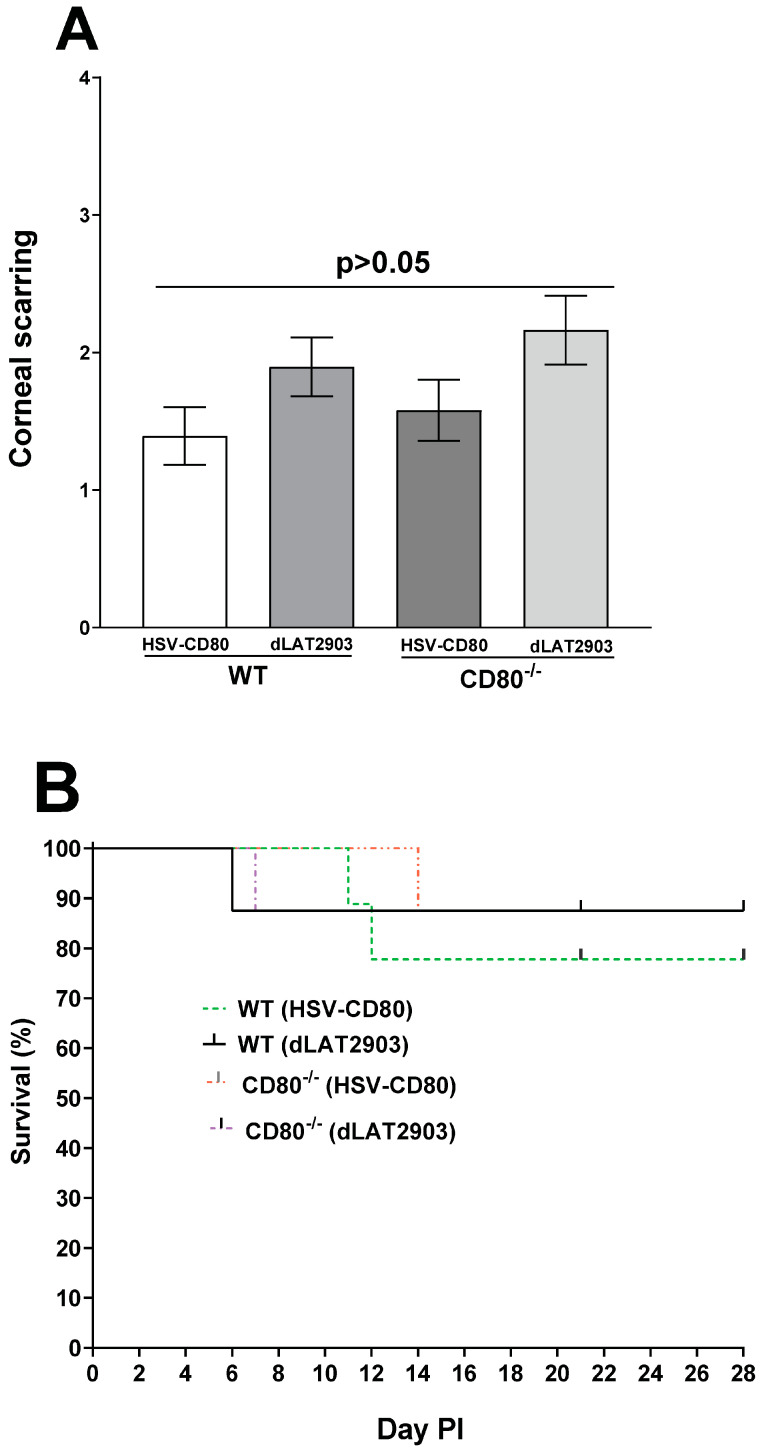
**Absence of CD80 or LAT does not affect survival and eye disease**. (**A**) Eye disease. Thirty-two eyes from and WT (HSV-CD80 virus-infected), CD80^-/-^ (HSV-CD80 virus-infected), and CD80^-/-^ (dLAT2903 virus-infected) mice and twenty-six eyes from WT (dLAT2903 virus-infected) mice were used to measure corneal scarring (CS). The severity of CS in mouse corneas was examined in all groups by slit lamp biomicroscopy. The CS severity was scored on day 28 PI from three independent experiments. All the infected mice groups were not significantly different from one another (*p* > 0.05). CS is based on 48 eyes for the WT mice infected with HSV-CD80 virus and the CD80^-/-^ mice infected with HSV-CD80 and dLAT2903 viruses, while CS in the WT mice infected with dLAT2903 virus is based on 46 eyes; and (**B**) mice survival. The mice were ocularly infected as described above. Survival of the WT and CD80^-/-^ mice was monitored over a 28-day period after infection. There were no differences in survival among all the infected mice groups (*p* > 0.05). Survival is based on three independent experiments.

**Monitoring the effects of the absence or presence of CD80 on eye disease and survival of infected mice.** CD80 is a critical factor in maintaining survival or clinical diseases in the host as it is the adaptive immune response playing the most fundamental role in HSV-1 infection [34]. To evaluate the effects of CD80 expression on corneal scarring (CS) and mouse survival, WT and CD80^-/-^ mice were ocularly infected with 2 × 10^5^ PFU/eye of dLAT2903 and HSV-CD80 viruses as above. We recorded mouse survival and CS in the surviving mice on day 28 PI. CS in the surviving mice was similar in the WT and CD80^-/-^ mice infected with HSV-CD80 or dLAT2903 virus (Figure 8A, *p* > 0.05). Out of 29 WT mice infected with HSV-CD80 virus, 24 mice survived (83%); 24 out of 28 (86%) WT mice infected with dLAT2903 virus survived ocular infection; similarly, 24 out of 27 (89%) CD80^-/-^ mice infected with HSV-CD80 virus survived; and 24 out of 25 (96%) CD80^-/-^ mice infected with dLAT2903 virus survived ocular infection. These differences in the levels of survival among these four groups were not statistically significant (Figure 8B, *p* > 0.05). Thus, the absence of CD80 in CD80^-/-^ mice or the expression of CD80 by HSV-1 did not significantly alter survival in infected mice.

**Figure 9:**
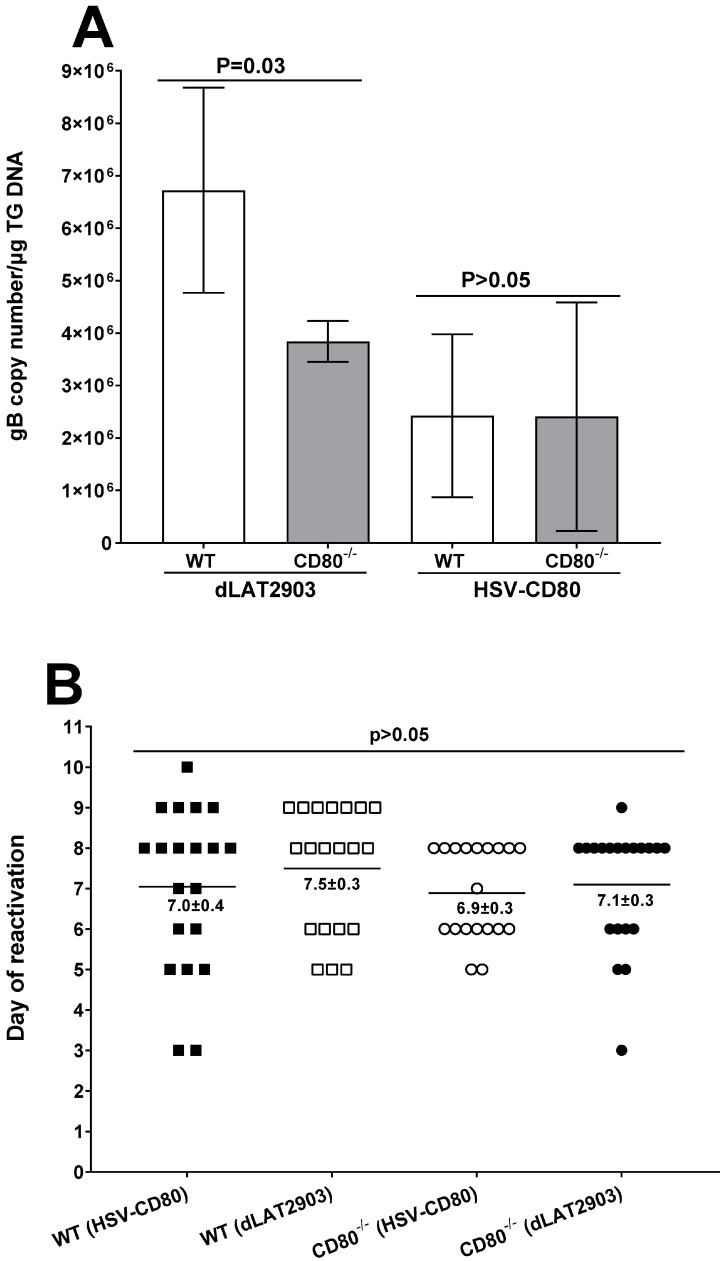
**The latent gB DNA and duration of explant reactivation following ocular infection of WT and CD80^-/-^ mice.** (**A**) gB DNA copy numbers in the latent TG. Forty TG from each infected mice group were isolated on day 28 PI. Expressions of gB DNA were determined using qPCR, and gB copy numbers were measured as described in Section 2. gB DNA copy numbers were higher in the WT mice infected with dLAT2903 virus compared with the CD80^-/-^ mice infected with dLAT2903 virus **(*p* = 0.03).** No differences were detected in the WT and CD80^-/-^ mice infected with HSV-CD80 virus (*p* > 0.05); and (**B**) explant reactivation in the latent TG. On day 28 PI, the TG from the infected WT and CD80^-/-^ mice were isolated and incubated in 1.5 mL of tissue culture media at 37 °C, and the presence of infectious virus was monitored as described in Section 2. The results are shown as the number of the TG that reactivated daily. Each point represents mean reactivated TG ± SEM of 20 TG from each mice group infected and from two independent experiments. There were no differences in reactivation (*p* > 0.05).

**Absence or presence of CD80 affects levels of latency but not reactivation.** To assess whether CD80 plays a role in latency, gB expression was determined in the WT and CD80^-/-^ mice infected with 2 × 10^5^ PFU/eye of dLAT2903 and HSV-CD80 viruses on day 28 PI. In this study, we used gB DNA levels rather than LAT expression because both HSV-CD80 and dLAT2903 viruses lack LAT expression [23,24]. gB DNA was significantly higher in the WT mice infected with dLAT2903 virus compared with the CD80^-/-^ mice infected with dLAT2903 virus, and these differences were statistically significant (Figure 9A, *p* = 0.03). In contrast, levels of latency were similar between the WT and CD80^-/-^ mice infected with HSV-CD80 virus (Figure 9A, *p* > 0.05). These results suggest that the absence of CD80 decreases levels of latency in latently infected mice, while CD80 expression by HSV-CD80 virus alter these differences.

To further analyze the effects of CD80 absence on explant reactivation, the TG from four groups of the infected mice were isolated on day 28 PI and monitored for the presence of infectious virus by explant reactivation as described in Section 2. The average time to reactivation in the WT mice infected with HSV-CD80 virus was 7.0 ± 0.4 days; in the WT mice infected with dLAT2903 virus, it was 7.5 ± 0.3 days; in the CD80^-/-^ mice infected with HSV-CD80 virus, the time to reactivation was 6.9 ± 0.3 days; and in the CD80^-/-^ mice infected with dLAT2903 virus, it was 7.1 ± 0.3 days. Overall, no significant differences among the four groups were detected (Figure 9B, *p* > 0.05). These results suggest that the absence of LAT expression or overexpression of CD80 affected levels of latency compared with the WT mice infected with parental virus, but not reactivation.

## 4. Discussion

HSV-1 infections are amongst the most frequent serious viral eye infections in the U.S. and are a major cause of viral-induced blindness [35,36,37]. In the U.S., approximately 30,000 people suffer recurrent ocular HSV episodes annually, requiring doctor visits, medication, and in severe cases, corneal transplants [35,36,37,38,39,40]. It is estimated that 70–90% of American adults have antibodies to HSV-1 and/or HSV-2, and about 25% of these individuals have clinical symptoms upon routine clinical inquiry [35,36,37,38,39,40], with HSV-1 being responsible for >90% of ocular HSV infections. A significant proportion (15–50%) of primary genital herpes is caused by HSV-1, and recent studies indicate that the proportion of clinical first-episode genital herpes due to HSV-1 is increasing [41,42,43]. Despite the seriousness of recurrent ocular herpes, no drug or vaccine has been approved by the FDA for prevention of ocular recurrences. Current use of antivirals and/or corticosteroids is limited as the virus is usually asymptomatic in early stages of infection; therefore, it becomes challenging when there are periodic outbreaks, often with lesions forming in infected individuals as a result of virus reactivation.

HSV-1 is an immunopathological disease, and combating inflammation is highly critical in controlling the pathogenesis. It is well known that T cells are the main orchestrators of the disease severity but can also include non-lymphoid cells, particularly neutrophils and macrophages as previously described [24,29,44,45]. To effectively target the disease severity, T cells need to be regulated. One approach could be to increase Treg cell population to suppress immune responses over T effectors, as shown before [46,47]. Another approach could be to tweak the activation source for effective T cell function and differentiation, for which we have studied the role of CD80 in our published reports [20,21,28,48]. Recently, we have shown that the absence of CD80 in CD80^-/-^ following infection with WT HSV-1 strain McKrae resulted in reduced virus replication in the eyes of infected mice compared with control WT mice, and the absence of CD80 significantly delayed virus reactivation despite the CD80^-/-^ and WT mice having similar levels of latency [28]. In contrast to our previous study in which we used WT McKrae, a LAT-plus virus, in our current study we used dLAT2903, a LAT-minus virus, as well as HSV-CD80, which is similar to dLAT2903 except it expresses CD80 under the LAT promoter. Thus, our current hypothesis was to examine if exogenously expressed CD80 by HSV-CD80 can compensate for the absence of CD80 and LAT in infected mice. Therefore, we compared WT and CD80^-/-^ mice after infection with HSV-CD80 and its parental control dLAT2903 virus.

Our results suggest that the absence or presence of CD80 had no effect on viral titers in the CD80^-/-^ mice or the infected control WT mice. This is in contrast to our recently published study using WT McKrae showing that ocularly infected CD80^-/-^ mice had lower virus replication than control WT mice [28]. This discrepancy between our current study and previous study suggests that exogenous CD80 with HSV-CD80 virus compensates for the lack of CD80 in CD80^-/-^ mice, and, therefore, the presence of CD80 in both WT and CD80^-/-^ mice does not have any effect in primary virus replication, and only CD80 absence controls virus replication in infected mice, as we have reported previously [28]. However, we do not believe that the absence of LAT in both HSV-CD80 and dLAT2903 viruses contributed to these differences. In our previous study, we showed that overexpression of CD80 led to increased levels of latency in the TG of latently infected mice compared to its parental control virus [27]. We found that levels of latency in the TG of CD80^-/-^ mice were significantly reduced as compared with WT mice infected with dLAT2903 virus, and levels of latency were not significantly different between WT and CD80^-/-^ mice infected. These results suggest that lack of CD80 in CD80^-/-^ mice following infection with dLAT2903 virus affects levels of latency in the TG of latently infected mice, while exogenous CD80 expression by HSV-CD80 virus restores levels of latency to that of WT mice (Figure 9A).

In our previous study [27], we have shown that CD80 has a detrimental role in increasing corneal scarring by increasing the recruitment of CD8^+^ T cells and activation; however, in our current report, the absence of CD80 had no effect on eye disease, and at the same time CD8^+^ T cells were not significantly different among all the experimental groups. Therefore, the absence of CD80 plays a protective role in HSV-1 infection by suppressing CD8^+^ T cell recruitment and, hence, reducing the tissue damage. To follow up with our previous study [28], we screened various gene expressions to measure inflammatory responses in the infected mice. We used a customized gene expression panel (see Section 2) to evaluate the effects of the absence of CD80 expression or overexpression of CD80 during HSV-1 primary infection. The innate cell expressions of many genes involved in HSV-1 infectivity were screened using qRT-PCR. Not much significant correlation was observed between HSV-1 pathogenicity and expressions of CD8, CD11c, NK1.1, LyG6, IL-1α, IL-1β, IL-2, IL-4, IL-6, IFNα2A, IFNβ, IL-12α, IL-12β, CD80, CD86, CD28, CD1d, perforin, GrzB, PD-L1, CD45, and TNF-α. In the WT mice infected with dLAT2903, CD4^+^ T cell expression in the TG on day 3 PI was increased in comparison to the CD80^-/-^ mice infected with HSV-CD80 (Figure 3C; *p* < 0.05). Also, on day 5 PI, in the TG, we saw increased F4/80 expression in the WT mice infected with HSV-CD80 in comparison to the WT mice infected with dLAT2903 (Figure 3F; *p* < 0.05). CD80 has been reported to suppress immune cells [49,50] and also to play a part in increasing inflammatory cascades, as reported in a polymicrobial sepsis model [51]; however, in our current study, on day 7 PI, in the CD80^-/-^ mice infected with dLAT2903 virus, the corneas of the infected mice displayed enhanced IFN-γ expression as compared to all other groups (Figure 5A; *p* < 0.05). These results suggest that the level of inflammation increases in the absence of CD80 expression after infection with dLAT2903 virus in the corneas but not the TG of infected mice. The absence of both LAT and CD80 in CD80^-/-^ mice infected with dLAT2903 virus could potentially lead to more inflammatory cytokine secretion as previously reported by [52].

The results of this study demonstrate that suppression of CD80 does have a therapeutic effect on reducing levels of latency. It is well established that HSV-1-induced corneal disease is associated with HSV-1 recurrences, and, thus, lower levels of latency may contribute to protection of the host from HSK by reducing virus reactivation. Overall, in the absence of CD80, immune responses can become balanced and aid in protecting the host from side effects associated with ocular HSV-1 infection.

## 5. Conclusions

This study suggests that the absence of CD80 does not affect virus replication in the eyes of infected mice, but it enhances inflammation in the corneas of infected mice. However, the absence of CD80 reduced the levels of latency, and this reduction in the levels of latency was compensated by a recombinant HSV-1 expressing CD80 under the latency associated transcript (LAT).

## Data Availability

All data is available within the manuscript.
